# Efficacy of Siriraj, in-house-developed, frozen gloves for cold therapy reduction of chemotherapy-induced peripheral neuropathy in gynecological cancer patients: randomized controlled trial

**DOI:** 10.1007/s00520-022-06890-1

**Published:** 2022-02-11

**Authors:** Phreerakan Chitkumarn, Tharinee Rahong, Vuthinun Achariyapota

**Affiliations:** Department of Obstetrics and Gynecology, Faculty of Medicine Siriraj Hospital, Mahidol University, Bangkok, 10700 Thailand

**Keywords:** Chemotherapy-induced peripheral neuropathy, Cold therapy, FACT/GOG-Ntx, Gynecological cancer, Cryotherapy, Paclitaxel

## Abstract

**Objectives:**

The primary objective of this study was to investigate the efficacy of cold therapy in reducing paclitaxel-based, chemotherapy-induced, peripheral neuropathy (CIPN). The secondary objective was to establish the incidence of CIPN arising from paclitaxel administration.

**Materials and methods:**

The study enrolled gynecological cancer patients who were aged over 18 years and receiving chemotherapy which included paclitaxel (175 mg/m^2^ every 3 weeks). The patients were allocated to control and cold-therapy groups by computer randomization. During paclitaxel administration, frozen gloves developed in-house by Siriraj Hospital were worn—with a cold pack inside—on both hands and both feet by the cold-therapy patients. The CIPN incidence was evaluated by FACT/GOG-Ntx (version 4) at each chemotherapy cycle and at the 1-month follow-up after treatment completion.

**Results:**

There were 79 patients (control arm, 40; study arm, 39). The CIPN incidences in the control and cold-therapy groups were 100% and 48.7%, respectively. CIPN was significantly decreased in the intervention group between the first cycle and the 1 month follow-up after chemotherapy cessation (*P* value < 0.001). Four patients discontinued the cold therapy due to pain, but there were no serious adverse effects due to the therapy.

**Conclusion:**

The Siriraj Hospital, in-house-developed, frozen gloves can reduce CIPN effectively as part of cold therapy for paclitaxel-based chemotherapy. The benefits of using the gloves are apparent from the first chemotherapy cycle to the 1-month, post-treatment follow-up assessment.

## Introduction

The number of gynecological cancer patients is gradually increasing. The cancer type and its stage determine the treatment, for example, surgery, radiation, or chemotherapy. Taxanes, especially paclitaxel, are very commonly used for the chemotherapeutic treatment of gynecological cancers, such as ovarian cancer, cervical cancer, and endometrial cancer. However, paclitaxel has many frequent side effects, including bone marrow suppression, alopecia, myalgia, nausea and vomiting, and peripheral neuropathy.

Chemotherapy-induced peripheral neuropathy (CIPN) is one common side effect of taxanes. The incidence rate of CIPN varies from 20 to 100%, and its severity depends on the cumulative dose of paclitaxel [1-3]. The symptoms can be either acute or chronic. Some patients develop acute symptoms after the first cycle of chemotherapy. Others, however, develop chronic symptoms after treatment completion, leading to disabilities and impaired quality of life [2]. CIPN affects the quality of life through the presence of pain and fatigue, impairment of cognitive function and mental health, and degradation of physical functioning [4]. Peripheral nerve damage can lead to either motor or sensory symptoms. The sensory symptoms are numbness, tingling, and burning pain, which eventually lead to motor symptoms [5]. Some symptoms may disappear after treatment discontinuation, but severe symptoms may persist for a prolonged period. The presence of CIPN necessitates discontinuation or dose reduction depending on severity, which consequently the treatment is not as effective as anticipated [1, 6].

Many studies have investigated the use of pharmaceutical and supplementary agents as well as alternative medicine for CIPN prevention and management. In Thailand, gabapentin and vitamin B complex are commonly prescribed for CIPN, despite the absence of a solid evidence base to support their use. In a study on ovarian cancer patients, gabapentin (900 mg a day) improved pain and neurological deficits in patients who developed peripheral neuropathy during carboplatin and paclitaxel administration. However, their quality of life did not change significantly [6]. The data are still inconsistent and limited; therefore, the American Society of Clinical Oncology clinical practice guidelines and review literature do not recommend CIPN treatment [7, 8]. In addition, a randomized, controlled trial examining whether vitamin B complex prevents CIPN found that the vitamin B complex could not significantly reduce CIPN more than a placebo [9]. Lastly, duloxetine was shown to significantly reduce CIPN in patients with gynecological cancer who received paclitaxel. However, the side effects of duloxetine were pronounced. They included somnolence, giddiness, nausea, constipation, dysgeusia, depression, and aggression, and they were particularly evident among elderly patients [10]. In addition, many trials studied acupuncture to reduce the incidence of CIPN. A recently published systematic review and meta-analysis concluded that although acupuncture was safe, symptom-alleviating effect on CIPN, however, could hardly be determined [11]. Like, electroencephalographic neurofeedback, yoga, therapeutic massage, their efficacy is still inconsistent. Further research is needed to understand their efficacy in preventing or treating the CIPN [4, 12].

Several studies have explored the effects of cold therapy, or “cryotherapy”, on the prevention of CIPN. A previous study, experimental cooling of hands of healthy subjects to 19 °C decreased blood flow to the palms about 53–60% [13]. The hypothesis is that if the nerve blood flow is reduced during paclitaxel infusion, the level of paclitaxel at the periphery will be decreased accordingly. However, the effectiveness of cold therapy is still inconclusive. In previous prospective studies, cold therapy significantly lowered the incidence of CIPN arising from the usage of paclitaxel [1, 3, 14]. The current study investigated the efficacy of frozen gloves developed in-house by Siriraj Hospital. The gloves were used during cold therapy that was administered to prevent the development of CIPN in gynecological cancer patients receiving paclitaxel.

## Materials and methods

### Patients

This randomized, controlled trial enrolled gynecological cancer patients (ovarian, cervical, endometrial, and uterine cancers) who received chemotherapy that included paclitaxel. The drug was administered at a dosage of 175 mg/m^2^ for 3 h every 3 weeks, concomitant with a platinum-based, or other, regimen. The study was conducted at the gynecological oncology ward of the Department of Obstetrics and Gynecology, Faculty of Medicine Siriraj Hospital, Mahidol University, Bangkok, Thailand, between May 2019 and November 2020. To be eligible for the intervention in this study, participants needed to be aged 18 years or older. Patients were not eligible if any subjective peripheral neuropathy symptoms were present before the chemotherapy initiation. They were also ineligible if they had potential peripheral neuropathy due to a prior taxane- or platinum-based chemotherapy regimen with disease recurrence within 1 year of that treatment’s cessation. Also, excluded were participants who had rheumatoid arthritis, diabetes mellitus, or peripheral vascular disease, or who were using drugs to treat peripheral neuropathy symptoms. Written informed consent for participation in this study was obtained from all eligible patients with a new diagnosis and those with a cancer recurrence that had occurred more than 1 year after prior chemotherapy. The enrolled patients were randomized during the intervention period into 2 groups: “cold-therapy” and “control.” Then their history chart was identified by a sticker to remind other medical staff not to prescribe any medication that would affect the CIPN. Patients in either group who discontinued chemotherapy early during the treatment (< 4 cycles) were excluded from the analysis. Before commencement of this research, its protocol was approved by the Siriraj Institutional Review Board (Si 267/2019). The trial was registered at the Thai Clinical Trials Registry (TCTR20210129001).

### Chemotherapy regimen

The details of the 3 chemotherapy regimens used were as follows: paclitaxel (175 mg/m2) intravenous (IV) drip in 3 h plus carboplatin (AUC = 5) IV drip in 1 h; paclitaxel (175 mg/m2) IV drip in 3 h plus cisplatin (50 mg/m2) IV drip in 6 h; paclitaxel (135 mg/m2 on day 1) IV drip in 3 h plus ifosfamide (1600 mg/m2 on days 1–3) IV drip in 6 h.

### Cold-therapy methodology

**Fig. 1:**
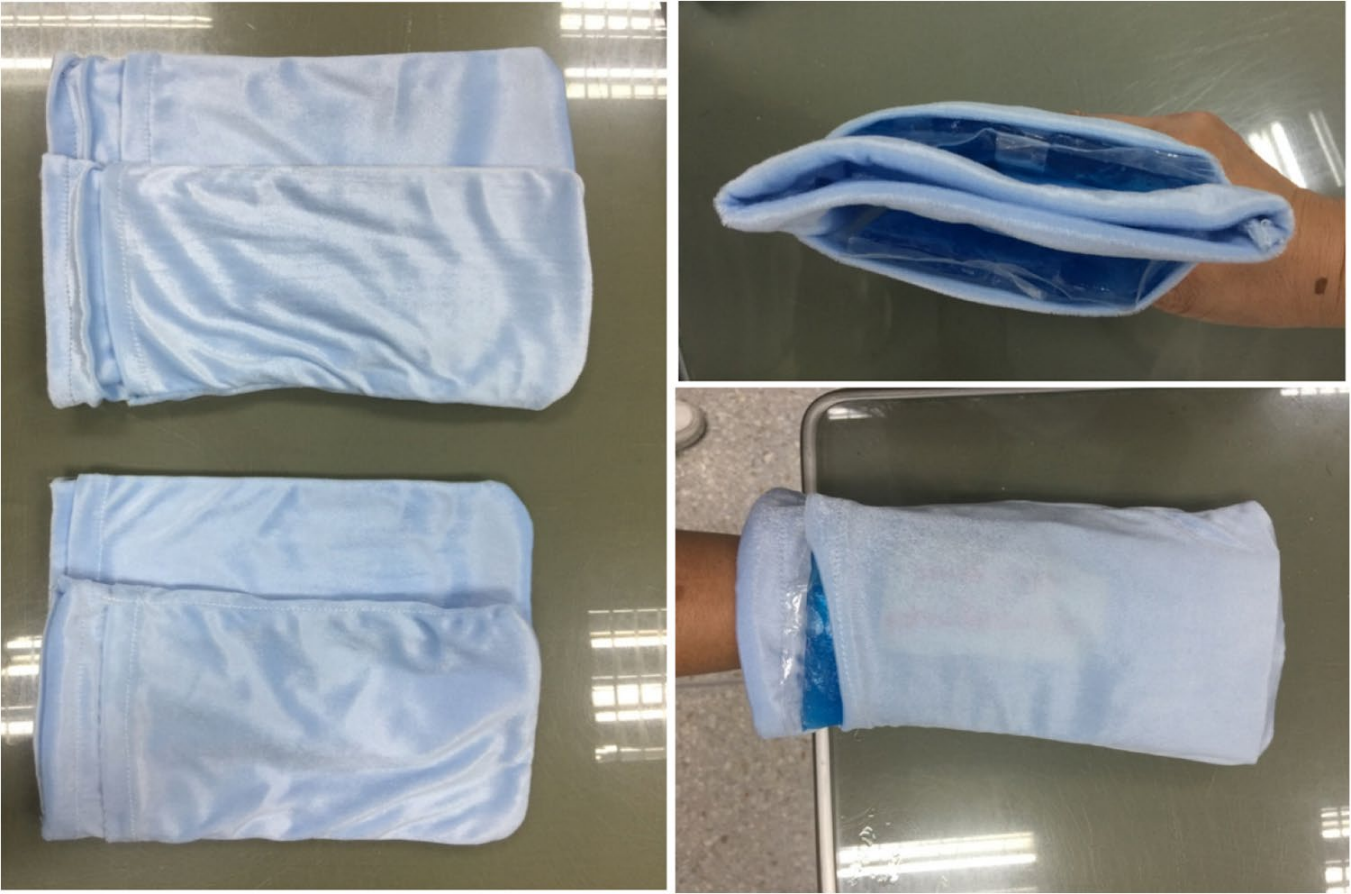
The Siriraj, in-house-developed, frozen gloves with 2 pieces of gel pack inside

The Siriraj, in-house, frozen gloves were developed using velvet fabric bags with 2 pieces of gel pack inside (Fig. 1). The gel pack (Nexcare; 3 M, St. Paul, MN, USA) was precooled in a refrigerator freezer before use. The velvet fabric bags were fitted over both hands and both feet for 3.5 h. They were left in situ from 15 min before to 15 min after the paclitaxel was administered. The fabric bags were changed every 15 min to maintain the temperature at 10 to 20 ºC by the surface probe of digital hygro-thermometer.

### Evaluation criteria and timing

The severity of CIPN was evaluated by questionnaire, using the Thai-version of the Functional Assessment of Cancer Therapy/Gynecologic Oncology Group-Neurotoxicity (FACT/GOG-Ntx; version 4). This health-related, quality-of-life questionnaire targets neurotoxicity-related chemotherapy treatment. It is comprised of 11 items, and patients report their sensory, motor, and auditory symptoms on a 5-point Likert-type scale ranging from 0 (not at all) to 4 (very much). The total score range is 0 to 44, with a high score signifying less neurotoxicity. The clinically significant development of CIPN was defined as a decrease in the FACT/GOG-Ntx score by > 10% between 2 consecutive time points [15, 16]. The evaluations for CIPN symptoms were conducted for up to 6 chemotherapy cycles, and a further evaluation was made at 1 month after the course finished.

### Primary and secondary objectives

The primary objective was to determine the efficacy of the Siriraj, in-house-developed, frozen gloves when used during cold therapy to prevent the development of CIPN in gynecological cancer patients receiving paclitaxel. The secondary objective was to establish the incidence of CIPN resulting from paclitaxel administration.

### Sample size calculation

According to a previous study, the incidences of CIPN in cold-therapy and control groups were 8% and 34%, respectively [1]. Using an alpha of 0.05 and a power of 80% to detect differences between the 2 groups, a sample size of 38 cases was calculated for each group. After allowing for an estimated 20% dropout rate, 47 cases were set for each arm.

### Statistical analysis

Statistical analyses were performed using IBM SPSS Statistics for Windows (version 27; IBM Corp., Armonk, NY, USA). The distribution of data was tested for normality using the Kolmogorov–Smirnov test. Descriptive data were summarized as mean (± standard deviation [SD]), median (with interquartile range [IQR]), or frequency and percentage. Differences between the cold-therapy and control groups were determined by using the Student’s *t*-test, Pearson’s chi-squared test, or Wilcoxon Rank Sum test, as appropriate. Linear mixed modeling analysis was conducted to determine the differences in the mean FACT/GOG-Ntx scores of the cold-therapy and control groups over each chemotherapy cycle. *P* values of less than 0.05 were deemed statistically significant.

## Results

**Fig. 2:**
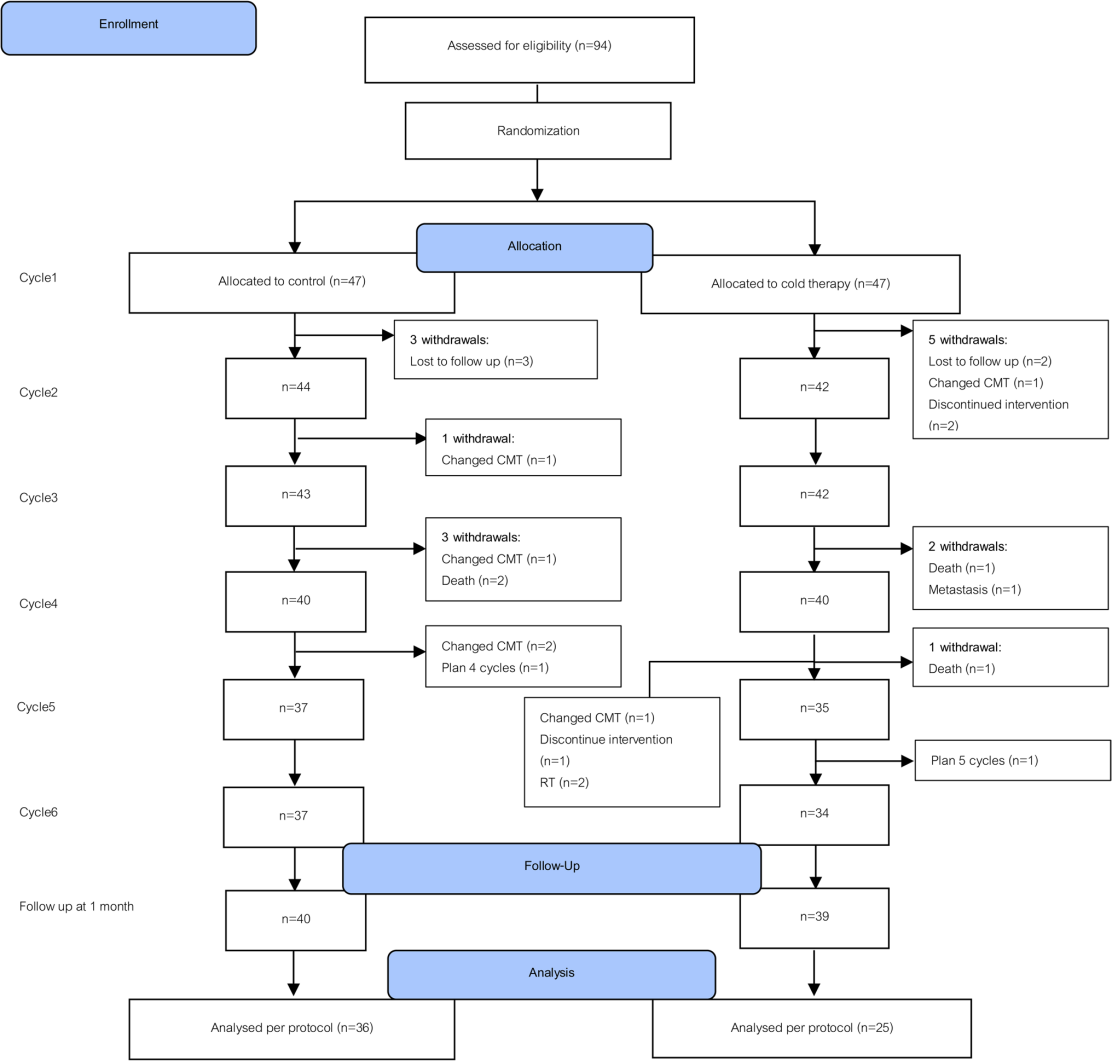
Consort flow of the participant’s diagram

In all, 79 gynecological cancer patients received paclitaxel-based chemotherapy (40 in the control group, and 39 in the cold-therapy group). The consort flow of the participants is illustrated in Fig. 2. There were no statistically significant differences in the baseline data of the 2 groups (age, body mass index, body surface area, type of cancer, cumulative dose of paclitaxel and of platinum, total cycles of chemotherapy, and FACT/GOG-Ntx score; Table 1).

**Table 1:** Characteristics baseline data of participants

Characteristics	Mean ± SD or *n* (%)		*p* value
	Control (*n* = 40)	Cold therapy (*n* = 39)	
Clinical			
Age (year)	55.45 ± 13.21	57.59 ± 11.23	0.441
Body mass index (kg/m^2^)	24.20 ± 5.62	22.76 ± 4.29	0.205
Body surface area (m^2^)	1.55 ± 0.20	1.53 ± 0.17	0.520
Cancer			
Ovary	25 (62.5%)	21 (53.8%)	0.283
Endometrium	11 (27.5%)	8 (20.5%)	
Cervix	3 (7.5%)	9 (23.1%)	
Sarcoma	1 (2.5%)	1 (2.6%)	
Platinum			
Cisplatin	3 (7.5%)	4 (10.3%)	0.553
Carboplatin	37 (92.5%)	34 (87.2%)	
Other (Ifosfamide)	0 (0%)	1 (2.6%)	
Cumulative dose of paclitaxel (mg)	1569.00 ± 215.08	1534.10 ± 241.64	0.500
Total cycles per patient	5.88 ± 0.21	5.81 ± 0.33	0.978
Baseline FACT score (Pre-chemotherapy)	43.58 ± 0.93	43.44 ± 1.27	0.580

Data are mean ± SD, or number (%)

There was a statistical difference in the CIPN incidences of the 2 groups: 100% for the control group, versus 48.7% for the cold-therapy group (*P* < 0.001; Table 2). The difference was significant with the initial chemotherapy cycle: for the cold-therapy group, the incidence was 15.4% (6/39), while in the control group, it was 35% (14/40; *P* = 0.045; Table 3). As a result, the higher the number of chemotherapy cycles administered was, the greater the statistical difference between the CIPN incidences of the 2 groups became (*P* < 0.001). At the 1-month follow-up session following the completion of the chemotherapy, the effectiveness of the cold therapy continued to be apparent. The CIPN incidence of the cold-therapy group (25.6%) was significantly lower than that for the control group (100%; *P* < 0.001; Table 3).

**Table 2:** Incidence of CIPN in each group

CIPN	Control (*n* = 40)	Cold therapy (*n* = 39)	*p* value
Yes	100% (40/40)	48.7% (19/39)	< 0.001
No	0% (0/40)	51.3% (20/39)	

^*^CIPN = chemotherapy-induced peripheral neuropathy

**Table 3:** Score of FACT/GOG-Ntx questionnaire and incidence of CIPN in each cycle of chemotherapy between control and cold therapy groups

	Control	Cold therapy	*p* value
Cycle 2			
FACT score (mean ± SD)	39.4 ± 3.88	41.44 ± 2.90	0.01
% Change of FACT score (mean ± SD)	− 9.59 ± 8.54	− 4.59 ± 6.30	0.004
% CIPN (*n*)	35% (14/40)	15.4% (6/39)	0.045
Cycle 3			
FACT score (mean ± SD)	37.92 ± 3.44	40.23 ± 3.57	0.005
% Change of FACT score (mean ± SD)	− 12.94 ± 7.89	− 7.28 ± 8.92	0.004
% CIPN (*n*)	57.5% (23/40)	28.2% (11/39)	0.009
Cycle 4			
FACT score (mean ± SD)	34.30 ± 4.95	40.87 ± 4.73	< 0.001
% Change of FACT score (mean ± SD)	− 21.29 ± 11.25	− 5.81 ± 11.55	< 0.001
% CIPN (*n*)	77.5% (31/40)	17.9% (7/39)	< 0.001
Cycle 5			
FACT score (mean ± SD)	34.03 ± 5.43	40.97 ± 3.71	< 0.001
% Change of FACT score (mean ± SD)	− 21.85 ± 12.39	− 5.55 ± 9.92	< 0.001
% CIPN (*n*)	89.2% (33/37)	28.6% (10/35)	< 0.001
Cycle 6			
FACT score (mean ± SD)	31.05 ± 5.75	40.44 ± 4.28	< 0.001
% Change of FACT score (mean ± SD)	− 28.69 ± 13.04	− 6.92 ± 11.11	< 0.001
% CIPN (*n*)	94.6% (35/37)	23.5% (8/34)	< 0.001
Follow-up at 1 month			
FACT score (mean ± SD)	30.13 ± 5.13	40.64 ± 5.70	< 0.001
% Change of FACT score (mean ± SD)	− 30.86 ± 11.75	− 6.33 ± 13.78	< 0.001
% CIPN (*n*)	100% (40/40)	25.6% (10/39)	< 0.001

Data are mean ± SD, or number (%), *CIPN* = chemotherapy-induced peripheral neuropathy

**Fig. 3:**
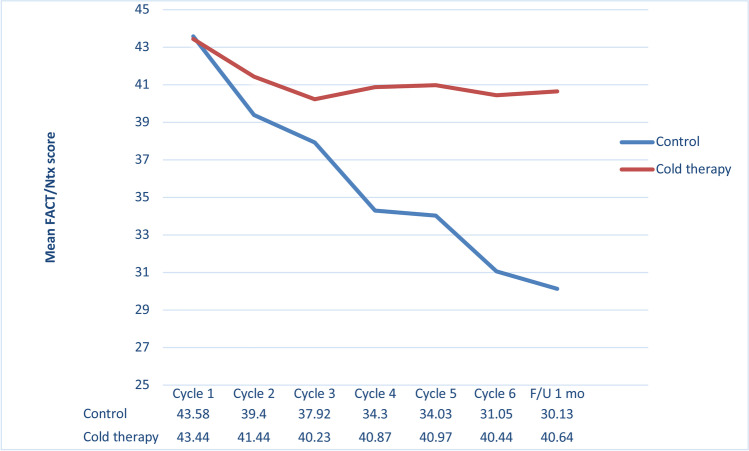
Mean FACT/GOG-Ntx questionnaire score in each cycle of chemotherapy and at the follow-up period between control and cold therapy group

The score for FACT/GOG-Ntx represented the severity of symptoms of CIPN and hence, the quality of life of the patients. After receiving the first chemotherapy cycle, the cold-therapy group’s score was significantly better than that for the control group (41.44 ± 2.90, versus 39.4 ± 3.88; *P* = 0.01). After completing the chemotherapy, at the 1-month review, the quality of life of the cold-therapy patients was still far superior to that of the control group (40.64 ± 5.70, versus 30.13 ± 5.13; *P* < 0.001; Table 3 and Fig. 3).

Four patients discontinued the cold therapy because they could not tolerate the discomfort caused by the low temperatures used. Apart from that, there were no substantial adverse effects, such as localized eczema or frostbite.

## Discussion

CIPN is one of the troublesome, common, dose-limiting complications related to neurotoxic-chemotherapy agents, especially platinum and taxanes, commonly used with gynecological cancer patients. Taxane-based therapy can induce paresthesia, numbness, and pain in a stocking-and-glove distribution (i.e., distal arms and legs) [17]. These symptoms affect the cancer-treatment process, necessitating discontinuation of the chemotherapy or, at least, a decrease in the paclitaxel dose. The severity of these symptoms depends on the cumulative dose of paclitaxel. Many pharmaceutical agents and supplements have been investigated to establish their efficacy in preventing or treating CIPN. Complementary and alternative medicines (for example, acupuncture) have been widely studied to explore their CIPN prevention and treatment potential. However, comprehensive scientific data in support of their use are still limited [18]. One alternative treatment modality, cold therapy (also termed cryotherapy), is a choice for CIPN management. It induces decreased blood flow to the target areas by vasoconstriction. In our recent investigation, we demonstrated a high incidence of CIPN as well as promising outcomes of our in-house-developed frozen gloves in lowering the CIPN rate.

In other studies, the incidence of CIPN varied from 20 to 100% of the patients who received paclitaxel [1-3]. In a comprehensive review, the author concluded that the rate was 68.1% when measured 1 month after the neurotoxic-chemotherapy completion, 60% at 3 months, and 30% at 6 or more months [19]. In another recent work, the incidence was 100% for the control group and 48.7% for the cold-therapy group. The difference in the CIPN rates was striking from the completion of the first-cycle chemotherapy to the 3-month, post-treatment, follow-up session. A similar finding was observed in the work of Sato et al. They investigated the use of cold therapy for gynecological cancer patients receiving tri-weekly, paclitaxel-based chemotherapy. Their results showed that there was a significantly lower incidence of peripheral neuropathy greater than grade 2 (under the Common Terminology Criteria for Adverse Events [CTCAE] grading system) for the cold therapy patients than for the controls [1]. Shigematsu and colleagues [14] demonstrated a reduction in the occurrence of CIPN when cold therapy was used for breast cancer patients receiving weekly paclitaxel. When assessed by the FACT/GOG-Ntx questionnaire, the CIPN rate for the cold group was significantly lower than that for control group (41% vs. 73%; *P* = 0.03). The incidence of peripheral neuropathy (either sensory or motor) ≥ 2 under the CTCAE system was also significantly lower for the cold-therapy group. The outcomes of our study might be comparable to those of the earlier investigations because of our strict inclusion and exclusion criteria relating to the presence of peripheral neuropathy before receiving chemotherapy.

The key to success with cold therapy is achieving and maintaining the optimal surface temperature at the hands and feet. To ensure that vasoconstriction would decrease the blood flow to the hands and feet, we maintained the temperature between 10 ºC and 20 ºC. A probe temperature was attached at the surfaces of the hands and feet to allow constant monitoring of their temperatures. However, some studies could not demonstrate the efficacy of cold therapy in reducing CIPN. In a multicenter, randomized, controlled trial, Beijers and coauthors investigated the efficacy of wearing commercial frozen gloves (Elasto-Gel Hypothermia TM-7006; Taureon, Nootdorp, Netherlands) to prevent CIPN during taxane treatment for breast and colorectal cancer. They concluded that there were no differences in the CIPN subscales of the cold-therapy and control arms [20]. It should be noted that the chemotherapy regimens employed in that study were heterogeneous. The taxanes used comprised oxaliplatin, paclitaxel, and docetaxel, with the majority of the participants (60%) in each arm receiving oxaliplatin. Oxaliplatin-induced neurotoxicity symptoms include cold-intolerance, discomfort, and muscle cramps. Furthermore, 34% of the frozen gloves were discontinued before the end of treatment. Since this is a high dropout rate, the interpretation of the results might be prejudiced. Moreover, as that research team did not measure skin surface temperature, it may have been higher than the optimal level.

One strength of the present investigation was that, as it was a randomized controlled trial, allocation bias and confounding factors were minimized. However, some selection bias may still occur in the patients in the control arm or in the patients who declined to participate. In addition, the temperature of the iced-gloves was well controlled, so the efficacy of the iced-gloves was reliable. Moreover, the FACT/GOG-Ntx scoring system was a feasible, reliable, and validated instrument for the assessment of CIPN. It is also sensitive to meaningful clinical distinctions and changes over time [21]. Lastly, we developed an in-house frozen glove which is inexpensive, safe to use, and as effective as commercial frozen gloves in terms of preventing CIPN. At Siriraj, frozen gloves costs approximately $5 per glove, a set of 4 gloves for both hands and both feet costs $20. This compares very favorably with the typical charge of US $80 for one commercial frozen glove, and $320 for a set of 4 gloves. The temperature range of the Siriraj frozen gloves was 10 ºC to 20 ºC, which was generally safe for the patients [1, 22]. However, 4 patients stopped the cold therapy because they could not tolerate the discomfort caused by these temperatures. Otherwise, there were no major adverse effects, such as localized eczema or frostbite. A systematic review of 11 trials also suggested that there are no serious adverse events arising from cryotherapy [12].

One limitation of this study was the method of CIPN assessment. Although the FACT/GOG-Ntx questionnaire was validated, the questionnaire is a subjective report in that it depends on patients’ experiences. Hence, it is less reliable than other assessment methods, such as the objective scores obtained with CTCAE (version 5) or the Total Neuropathy Score. The latter scoring system not only includes objective measures (such as pin prick, vibration threshold, and nerve conduction) but, importantly, a quality of life assessment [23]. The second limitation was the short follow-up period. CIPN can persist for a long time, so the follow-up period should be extended to 6 months to 1 year after chemotherapy. Another was that the current study was unblinded. Further study could put cold instead of ice gel packs in the control group to blind patients or health care providers to prevent bias. Finally, notwithstanding that cold therapy’s efficacy is good, we did not evaluate the patients’ satisfaction with using the frozen gloves. Hence, our future studies will use assessment tools that comprise subjective and objective evaluations, blinded studies, as well as long follow-up periods.

## Conclusion

The Siriraj, in-house-developed, frozen gloves can reduce CIPN effectively as part of cold therapy for paclitaxel-based chemotherapy. The benefits are apparent from the first chemotherapy cycle to the 1-month, post-treatment follow-up assessment.

## Data Availability

N/A.

## Code availability

N/A.
